# Aquifer Thermal Energy Storage: Groundwater for Efficient Data Center Cooling in the United States

**DOI:** 10.1111/gwat.70084

**Published:** 2026-05-26

**Authors:** Upasana Pandey, Andrew J. Stumpf, Yu‐Feng F. Lin

**Affiliations:** ^1^ Illinois State Geological Survey, Prairie Research Institute University of Illinois at Urbana‐Champaign 615 E. Peabody Drive Champaign IL 61820; ^2^ Illinois Water Resources Center University of Illinois at Urbana‐Champaign 615 E. Peabody Drive Champaign IL 61820

## Abstract

Data centers are energy end users with the fastest growing need for electricity in the United States, mainly because of the rapid expansion of cloud computing and artificial intelligence (AI). A substantial portion of this electricity, between 10% and 40%, is used for cooling. As the number of data centers increases and the sector's energy demand continues to rise exponentially, there is an urgent need to explore the use of alternative energy systems that are more efficient and sustainable. This article explores aquifer thermal energy storage (ATES) as a technically feasible and currently underutilized solution for data center cooling in the United States. Previous case studies from Europe and assessments based in the United States are considered, and the potential of ATES for reducing electricity usage for data centers, which would reduce overall greenhouse gas emissions and support sustainable energy operations.

## Graphical abstract

As the demand for data centers continues to rise exponentially, there is an urgent need to explore sustainable and efficient alternatives to conventional cooling systems for data centers. This article explores aquifer thermal energy storage as a technically viable and underutilized solution for data center cooling in the United States.

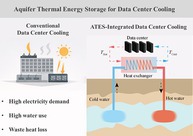

## Introduction

The United States currently hosts approximately 45% of the world's data centers (Cloudscene 2024), making it the global leader in installed artificial intelligence (AI) infrastructure, which has significant implications for national energy usage. In 2024, the energy demand of data centers was more than 4.4% of the country's total electricity generation, amounting to 180 terawatt‐hours (TWh) (IEA 2025). Driven by the rapid expansion of energy‐intensive AI hardware, this energy demand is projected to rise significantly by 2028, reaching between 6.7% and 12% of the total national electricity consumption, or roughly 325–580 TWh (Shehabi et al. 2024). As electricity becomes a critical factor, each additional megawatt of power capacity gains strategic value, supporting an estimated $25–$50 million in annual revenue and generating $10–$25 million in profit (Yusifov and Enriquez 2025).

The rapid development of data centers across the United States is not geographically uniform, but rather is driven by a combination of strategic, economic, and infrastructural factors (Greenstein and Fang 2022). Key considerations include (1) the costs of land, construction, and operations; (2) the availability of a stable power supply; (3) access to high‐capacity fiber‐optic networks (supporting low‐latency and high‐speed connectivity); and (4) proximity to end users or major data markets (Fawcett 2024). Collectively, these factors drive development decisions, making certain regions more attractive than others for large‐scale data center development. As a result of the high energy demands of data centers, they have become some of the largest single consumers of electricity in several states. For instance, at least six states (Iowa, Nebraska, North Dakota, Oregon, Virginia, and Wyoming) have reported that data centers require more than 10% of the total electricity available, with some regions accounting for as much as 25% of the share (Table 1) (Aljbour and Wilson 2024; Shehabi et al. 2024). The geographic concentration of data centers and their associated anomalous energy and water consumption illustrates how resource intensive they are (Li et al. 2023; Yañez‐Barnuevo 2025). Their localized impact is especially significant, with some jurisdictions delaying or canceling the permitting of any new facility because of limitations of the power grid (Spencer and Singh 2024).

**Table 1:** Data Center Energy Consumption in 2023 and Its Share of Statewide Electricity Use in Selected U.S. States (Aljbour and Wilson 2024)

State	Annual Energy Consumption from Data Centers (MWh)	Share of Total State Electricity Consumption (%)
Iowa	6,193,320	11.43
Nebraska	3,959,520	11.70
North Dakota	3,915,720	15.42
Oregon	6,413,663	11.39
Virginia	33,851,122	25.59
Wyoming	1,857,120	11.26

The large electricity demand of data centers is tied to its essential components such as information technology (IT) equipment, high‐performance servers, networking hardware, and power‐conditioning systems (Takci et al. 2025). The operation of these components generates substantial heat within the facility, which must be continuously removed to maintain safe operating conditions and prevent equipment overheating, necessitating the use of large cooling units (Ebrahimi et al. 2014; Nadjahi et al. 2018). In many facilities, IT equipment alone represents roughly half of total energy use, while cooling demands contribute close to 40% (Zhao and Miyamoto 2021; Takci et al. 2025). As a result, cooling is an essential component of data center operations, and cooling systems require between 10% and 40% of the total electricity supplied (Ni and Bai 2017). Conventional cooling technologies used in data centers, such as mechanical vapor‐compression refrigeration, electric chillers, and air‐conditioning systems can produce significant carbon emissions when powered by fossil fuel‐based electricity (Alkrush et al. 2024; Dai et al. 2024). On top of that, conventional cooling technologies, such as dry coolers, air‐ and water‐cooled chillers, condensers, or other mechanical systems, ultimately exhaust heat into the atmosphere, which further exacerbates local microclimate issues (e.g., urban heat islands) (Socci et al. 2024). These systems typically rely on chillers and cooling towers that release waste heat to the ambient environment, resulting in the loss of potential waste heat recycling and a continued dependence on electricity and, in many cases, water resources (Li et al. 2023). Moreover, data centers further contribute to an increasing water footprint through both direct cooling processes and indirect procurement water use (Xiao et al. 2025). Water‐related impacts of data center have been documented in several regions of the United States, highlighting the need for careful protection and management of local water resources in areas undergoing data center development (Volzer 2025). These limitations become more severe in hot climates or water‐stressed regions, where higher ambient temperatures or limited water availability require more energy‐ or resource‐intensive cooling solutions (Karimi et al. 2022). As overall electricity use in data centers continues to rise, the corresponding demand for cooling is expected to increase as well. Thus, optimizing the cooling process is a critical focus for data centers owners, along with the need to improve energy efficiency and reduce GHG emissions (Zhou et al. 2024).

Aquifer thermal energy storage (ATES), a type of underground thermal energy storage (UTES) system, provides cooling (heating) by recovering stored cold (heat) from aquifers. ATES systems are developed to utilize the underground for cooling by extracting groundwater, which is at a near‐constant temperature, and which has the operational capacity to seasonally store and recover thermal energy. This approach can achieve higher overall efficiency than many conventional heating and cooling technologies (Ramos‐Escudero and Bloemendal 2022) and can decrease reliance on fossil fuel–based energy sources (Liu et al. 2025). In regions with favorable groundwater availability and geological conditions, ATES have been successfully deployed for large‐scale cooling and for recovering stored heat for subsequent heating applications in commercial and industrial facilities (Bloemendal et al. 2018). When integrated with data centers, ATES systems eliminate the need to discharge waste heat into the atmosphere and can help reduce associated local microclimatic impacts, such as urban heat island effects. Interest in ATES has grown further in recent years, largely due to its high thermal storage potential, comparatively small environmental footprint, and increasingly favorable economics (Schmidt et al. 2018; Matos et al. 2019). Globally, ATES deployment is dominated by northwestern Europe, with the Netherlands accounting for roughly 85% of all operating systems, and an additional 10% located in Sweden, Belgium, and Denmark (Fleuchaus et al. 2018; Stemmle et al. 2025b). Although ATES systems have been successfully developed across parts of Europe (Pellegrini et al. 2019), they remain largely underutilized in the United States, particularly for data center cooling.

ATES is increasingly being considered as a strategy for reducing the substantial energy costs associated with data centers (Fleuchaus et al. 2018; Liu et al. 2020). With data centers expanding rapidly in the U.S. and cooling demand putting additional pressure on local energy infrastructures, it is important to consider ATES as an alternative cooling technology that also allows waste heat to be stored and reused (Dvorak et al. 2020; Yuan et al. 2023). The aim of this article is to address current knowledge gaps regarding the potential role of ATES in improving data center thermal management in the U.S. context. The article provides an overview of ATES as a heating and cooling technology, and reviews existing studies and applications that have explored its use for data center cooling. Finally, it discusses the broader potential of ATES for data center cooling in the United States.

## ATES System Overview

**Figure 1:**
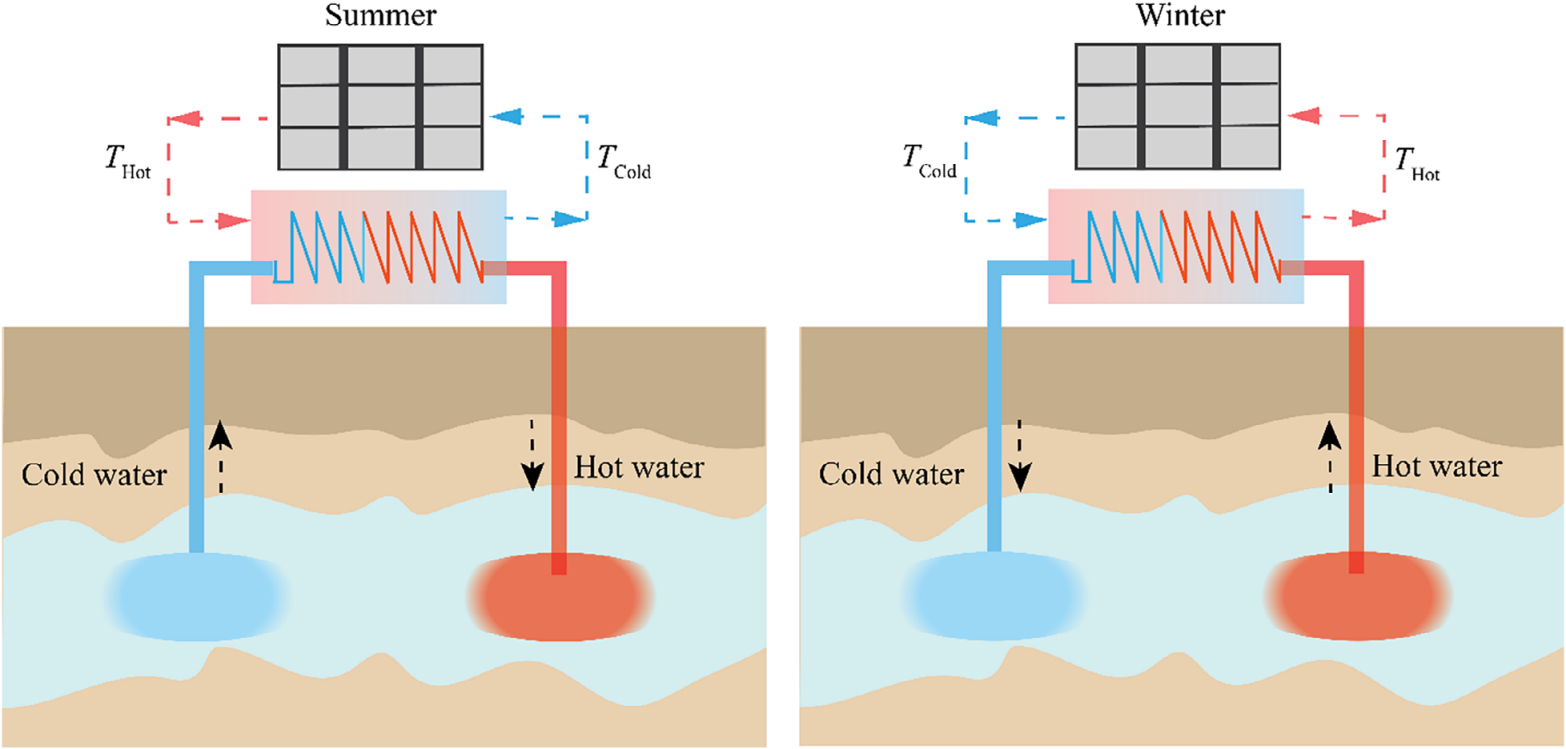
Operational schematic of an aquifer thermal energy storage (ATES) system illustrating operations in the summer (left) and winter (right).

Aquifers are subsurface porous media, typically composed of sand, gravel, and rock formations, whose pore spaces can hold groundwater (Rad and Fung 2016). These water‐bearing formations are well suited for thermal energy storage due to their large storage volume (Sanner 1999; Radwan and Humphrey 2023; Stemmle et al. 2025a). Groundwater temperatures within such formations generally remain stable and correspond closely to the local mean annual surface temperature (Paksoy and Beyhan 2015), making aquifers inherently suitable for thermal energy storage. ATES systems are commonly used for space heating and cooling of large building complexes and for integration into district heating (DH) and district heating and cooling (DHC) networks (Stemmle et al. 2025a). In cooling operation, groundwater is pumped from the aquifer and circulated through a heat exchanger, where it absorbs heat removed from the building. The warm water is then reinjected into a separate well positioned at an adequate distance from the extraction well to maintain hydraulic and thermal separation. The injected heat remains stored within the aquifer until it is required. During winter, warm groundwater is withdrawn for heating applications. In this mode, heat is transferred from the groundwater to the building through a heat exchanger, after which the cooled groundwater is reinjected into the aquifer through the other well. A schematic representation of this operational cycle is provided in Figure 1. Within an ATES system, wells are classified according to the temperature of the groundwater they store and supply, commonly referred to as the warm (or hot) well and the cool (or cold) well. Generally, they are not designated as “injection” or “production” wells because ATES wells operate bi‐directionally; each well can serve either as an extraction or an injection well, depending on whether the system is operating in heating or cooling mode (Jackson et al. 2024).

ATES systems may operate either with or without heat pumps, depending on system requirements. During the heating operation, heat pumps are used to further heat the extracted water before supplying it to the building heating distribution network and domestic hot water system (Abuasbeh and Palm 2025). During direct cooling operation (also known as passive cooling), the ATES system delivers cooling directly to the building cooling distribution network without using heat pumps (Jackson et al. 2024; Abuasbeh and Palm 2025). In this mode, electricity consumption is very low, resulting in a high system coefficient of performance (COP), attributed to the seasonal storage and subsequent utilization of previously recovered cooling energy (Jackson et al. 2024). In cooling operation with heat pumps (also known as active cooling), the heat pumps are used to provide cooling to the building cooling distribution network when direct cooling from the ATES is insufficient (Beernink et al. 2022; Abuasbeh and Palm 2025). In addition, hybrid ATES configurations that incorporate supplemental air‐ or water‐cooled chillers can further optimize the operation and control systems to achieve seasonal COPs of more than 25 (Schüppler et al. 2019).

For an ATES system to provide sustainable long‐term heating and cooling, it must be designed to maintain thermal balance, meaning that the quantities of heat and cold stored in the aquifer annually are approximately equal (Sommer et al. 2014). Maintaining this balance minimizes long‐term shifts in aquifer temperature and reduces the likelihood of thermal interference between warm and cold plumes, both of which can degrade storage performance and overall system efficiency (Sommer et al. 2014; Rostampour et al. 2019). In cases where annual heating and cooling demands are inherently mismatched, supplemental heating or cooling sources may be incorporated to restore thermal balance within the ATES system (Hoekstra et al. 2020).

ATES systems can enable the capture and reuse of waste heat and cooling within the same building or facility. ATES may also be configured to store waste heat or cooling from one source for use in another location, for example, excess industrial heat generated can be stored in the aquifer and utilized to supply space heating to nearby buildings in winter (Jackson et al. 2024). Common sources include industrial waste heat as well as renewable thermal resources such as excess solar thermal energy or geothermal heat (Wesselink et al. 2018; Stemmle et al. 2025a). This operational flexibility is particularly advantageous for data centers, where large quantities of recoverable waste heat and continuous cooling demand make ATES suitable with desired synergy.

**Figure 2:**
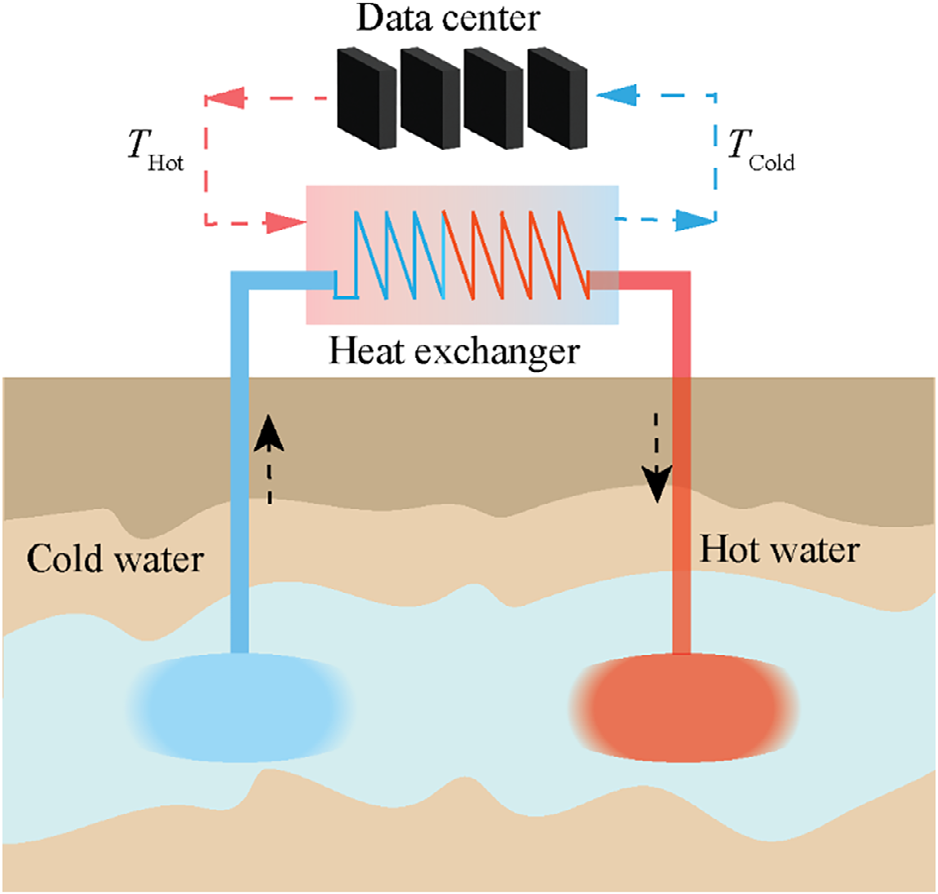
Schematic representation of an ATES system integrated with a data center. For data center cooling, cold groundwater is extracted from a dedicated cold well and circulated through a heat exchanger, where it absorbs waste heat from the data center. The heated groundwater is then reinjected into the aquifer via a separate warm well, thereby storing thermal energy in the subsurface. During heating operation, the flow direction is reversed and the stored heat is recovered from the warm well to supply heat to nearby facilities or to a thermal energy network (TEN), while the cool water is reinjected into the cold well.

When applied to data center cooling, the operational concept of ATES can be described as follows:

- Groundwater would be pumped from a well that is at ambient ground temperature to cool data center equipment using heat exchangers (Figure 2). After the heated water exits the data center, it is returned to the same aquifer in a second well and is stored until the winter season.
- In winter, when the air temperature is cooler, the warmer water in the second well should be extracted to heat nearby facilities (commercial and residential buildings). After the heat is exchanged, the cooled water should be pumped into the other well to charge (further cool) the aquifer used for data center cooling. To achieve long‐term thermal balance, ATES operation should be integrated with the thermal energy network (TEN) or nearby heat‐consuming facilities, such that the quantity of heat stored during data center cooling operation is appropriately matched to the available seasonal heating demand.

By separating the warm and cold thermal plumes for seasonal thermal energy storage, ATES systems effectively recycle the thermal energy, providing an energy‐efficient heating and cooling method.

ATES systems can be classified according to their storage temperature into low‐temperature ATES (LT‐ATES) and high‐temperature ATES (HT‐ATES) (Stemmle et al. 2025a). LT‐ATES systems are commonly charged during the warm season by injecting waste heat into the aquifer at temperatures up to approximately 25 °C (Fleuchaus et al. 2018, 2020), and often operate in combination with a heat pump. In contrast, HT‐ATES systems operate at significantly higher storage temperatures, typically exceeding 40 °C (Fleuchaus et al. 2018). When ATES is integrated with a data center, the waste heat produced by the IT and cooling infrastructure becomes the primary thermal input to the system. Data center operations can generate waste heat across a broad temperature range, depending on the cooling system used. When air‐side cooling or air‐to‐liquid cooling systems are used, waste heat temperatures typically range from approximately 15 °C–30 °C, whereas data centers using liquid‐cooling systems can produce waste heat at significantly higher temperatures, commonly within the range of 50 °C–60 °C (Yuan et al. 2023). Therefore, an ATES system coupled to a data center may be classified either as a low‐temperature ATES (LT‐ATES) or a high‐temperature ATES (HT‐ATES), depending on the selected data center cooling system.

The successful implementation of an ATES system requires that specific hydrogeological conditions be satisfied. Key criteria include adequate permeability and porosity of the aquifer, sufficient thickness (and therefore storage capacity), and an appropriate depth of the storage layer (Ramos‐Escudero and Bloemendal 2022; Shi et al. 2023). Low permeability can restrict achievable pumping rates and increase the energy demand of groundwater extraction (Chen et al. 2026). Whereas, excessively high permeability can promote unwanted convective transport within the aquifer, resulting in thermal losses and reduced recovery efficiency. In addition to hydrogeological properties, hydrochemical conditions play a critical role in ATES sustainable operation (Kim et al. 2023). Temperature changes may induce mineral precipitation, leading to scaling and corrosion, depending on groundwater chemistry (Opel et al. 2014). Thermal perturbations may also influence the native microbiological community within the aquifer (Kuznik et al. 2021). When integrating ATES with a data center, a crucial design challenge is ensuring an effective balance between the heat rejected to the aquifer and the heat recovered for external heating applications. As data centers are cooling dominated facilities with continuous year‐round cooling requirements, achieving this balance is essential for sustainable subsurface operation. Therefore, in addition to meeting hydrogeological requirements, careful attention to system integration and thermal distribution design is critical for ATES deployment in data center applications.

## ATES Applications and Discussion

The application of ATES systems dates back to the 1960s, when the first deployments were implemented in China primarily for industrial cooling purposes (Sanner 2016; Fleuchaus et al. 2018). Over the following decades, ATES technology was explored in several countries, gradually evolving from experimental systems to reliable seasonal thermal energy storage solutions. At present, worldwide experience with ATES is substantial, with the most extensive and mature deployment observed in Europe. In particular, the Netherlands has emerged as the global leader in ATES implementation, with more than 3000 operational systems (Bloemendal et al. 2023). This widespread adoption is largely driven by favorable hydrogeological conditions, such as shallow, sandy, and productive homogeneous aquifers, and a regulatory framework that actively supports subsurface thermal energy storage (Bloemendal et al. 2015; Drijver and Godschalk 2018; Jackson et al. 2024; Stemmle et al. 2025b). Additional European installations are concentrated in Sweden, Denmark, and Belgium (Possemiers et al. 2014; Nordell et al. 2015; Anibas et al. 2016; Jackson et al. 2024; Stemmle et al. 2025b). The scale and long‐term operation of these systems indicate that ATES is a well‐understood technology when deployed under geological conditions with little uncertainty along with supportive institutional frameworks.

In contrast, ATES development in North America, Asia, and other parts of Europe remains at a relatively early stage, despite the presence of regions with suitable aquifer conditions (Bloemendal et al. 2015; Liu et al. 2025). In the United States, early ATES research primarily took place between the 1970s and mid‐1980s, focusing on experimental and demonstration‐scale systems operating at low technology readiness levels (Fleuchaus et al. 2018; LBNL 2018). These early efforts, while limited in commercial impact, played a critical role in establishing the scientific and engineering foundations of ATES. One of the most notable early studies was conducted at Auburn University, Alabama, where an ATES system was tested with injection depths of approximately 61 m and storage temperatures ranging from 45 °C to 65 °C, classifying it as a high‐temperature ATES (HT‐ATES) system (Molz et al. 1979; Molz et al. 1983a). Operational challenges identified in this project included clogging of injection and extraction wells and reductions in hydraulic conductivity caused by clay particle migration and pore blockage. These findings highlighted the importance of detailed subsurface characterization and appropriate aquifer selection to achieve high thermal recovery (Molz et al. 1979). Further advances were achieved through an ATES design and field test site at the University of Minnesota, St. Paul, where storage temperatures between 80 °C and 115 °C were investigated (Hoyer et al. 1994). This study specifically highlighted the influence of groundwater chemistry on ATES design and operation, demonstrating that site‐specific operational strategies are essential for optimizing energy recovery. The findings contributed to refining thermal design approaches and operational control strategies for ATES systems under high‐temperature conditions (Hoyer et al. 1994).

Several U.S. demonstration projects also explored ATES as a cold storage technology to reduce peak cooling demand during summer periods. An example is the ATES system implemented in Tuscaloosa, Alabama, which was designed exclusively for cold storage (Melville et al. 1985). This system reportedly achieved a reduction of approximately 20% in peak cooling demand compared to conventional cooling systems (Melville et al. 1985). Unlike modern two‐well ATES configurations, the system stored chilled water in an aquifer and later recovered it for cooling, after which the water was discharged for irrigation or stormwater drainage rather than reinjected in the aquifer (Schaetzle and Brett 1989). A similar cold storage ATES demonstration was conducted at Stony Brook, New York, where the system was found to be technically feasible for campus cooling applications (Minor 1981; Kannberg 1982; Supkow and Shultz 1982). A more recent example of ATES deployed as a seasonal cold storage system in the United States is the installation at Stockton University, New Jersey (Smith et al. 2021). The system operates within a shallow (30–60 m depth) semi‐confined aquifer and was designed using six wells arranged as three cold wells and three warm wells, with the cold and warm well fields separated by approximately 300 m (Smith et al. 2021; Kumar et al. 2024). In contrast, the ATES system implemented in Melville, New York, was designed to provide both heating and cooling, although the building facility itself was cooling‐dominated (Marseille et al. 1993). As a result, auxiliary electric chillers were required to meet peak cooling loads, and a liquid desiccant system was incorporated to address humidity control challenges. Collectively, these early U.S. projects served as important stepping stones in the global development of ATES knowledge, informing best practices related to subsurface characterization, groundwater chemistry, system configuration, and operational optimization.

In recent years, ATES has gained attention in the United States as a potential approach to achieve energy efficiency and the need for seasonal energy storage. A notable example is the feasibility assessment conducted for the Ford site in St. Paul, Minnesota, which reported approximately 40% primary energy savings, a 35% reduction in CO_2_ emissions, complete elimination of cooling water consumption, and a 17% reduction in operational expenditures compared to a conventional system composed of centralized gas boilers and electric chillers (Worthington 2016). The elimination of cooling water consumption represents a significant advantage of ATES, particularly in the context of data centers, where consumptive water use has emerged as a significant concern. The study further concluded that more than 75% of the annual cooling demand could be met through direct cooling, supported by favorable climatic conditions, suitable hydrogeology, and relatively balanced thermal loads (Worthington 2016). ATES has also been investigated as a component of TENs in dense urban environments, such as Manhattan, New York (Fry et al. 2023). These studies emphasize the need for detailed subsurface characterization and advanced groundwater modeling to support reliable ATES design and operation in complex urban settings.

When considering ATES applications specifically for data center cooling, most operational systems remain concentrated in Europe. A prominent example is the Bonner Bogen development in Bonn, Germany, where a commercial low‐temperature ATES (LT‐ATES) system has been in continuous operation since 2009 (Stemmle et al. 2021). This system supplies heating and cooling to a mixed‐use district that includes office buildings, a hotel with a congress center, a medical center, and a data center (Stemmle et al. 2021). The Bonner Bogen ATES system is among Europe's largest heat pump installations. Six wells with depths ranging from 22 to 28 m serve a total usable floor area of roughly 60,000 m^2^ (Stemmle et al. 2021). During the heating season, 60–80% of the heat demand is met using warm aquifer storage in combination with heat pumps, with gas boilers available to cover peak loads during periods of very low outdoor temperatures (Mands et al. 2010). In summer, groundwater circulation is reversed to enable direct cooling, supported by refrigeration machines as required. The year‐round cooling demand of the data center results in elevated heat injection into the aquifer, emphasizing the importance of thermal balancing and long‐term storage management in ATES‐supported data center applications.

An example of ATES application for German data center cooling systematically evaluates the integration of ATES into the cooling systems of mid‐size data centers under German climatic and hydrogeological conditions, addressing both technical feasibility and economic performance (Drenkelfort et al. 2015). Using site‐specific weather data, aquifer properties, and cooling system configurations, the study analyzed ATES‐supported cooling operation for multiple representative locations (Berlin, Hamburg, and Munich) in Germany. The results demonstrate that ATES can significantly reduce the energy demand of data center cooling systems by enabling extensive use of seasonal free cooling, with reported electricity savings for cooling ranging from modest values in less favorable subsurface conditions to more than 20% in locations with thick, productive aquifers. Importantly, the analysis also demonstrates that ATES‐based cooling can be economically viable for data centers when supported by energy‐efficiency incentive programs, particularly in regions with higher electricity prices and suitable underground conditions (Drenkelfort et al. 2015).

Translating these European experiences to the U.S. context requires careful consideration of differences in climate, hydrogeology, regulatory structures, and cost factors. With groundwater temperatures between 10 °C and 15 °C observed at depths typically ranging from approximately 50 to 150 ft (15–45 m) in many regions of the United States (Dexheimer 1985), ATES offers a technologically and commercially proven alternative for data center cooling. More recently, the ATES concept has been extended to deeper and lower‐quality groundwater formations, such as brackish aquifers, under the designation of reservoir thermal energy storage (RTES) (Burns et al. 2020). These deep, geochemically evolved aquifers are hydraulically isolated from shallow freshwater sources and exhibit minimal natural groundwater flow (Burns et al. 2020). RTES systems are generally developed at depths ranging from approximately 150 m to more than 1000 m, with ambient reservoir temperatures that vary widely depending on depth and local geothermal conditions (Pepin et al. 2025). Deeper reservoir formations tend to exhibit elevated temperatures due to the natural geothermal gradient (Pepin et al. 2025), whereas at shallower depths, lower ambient reservoir temperatures often make the upper portions of the reservoir more favorable for cold energy storage (Nathenson and Guffanti 1988). A study conducted in Golden, Colorado demonstrated that such a system could meet cooling demand while storing cold water during the winter season (Oh et al. 2025). Reported system COP ranged from approximately 38 when coupled with dry coolers to as high as 54 when integrated with both dry coolers and heat recovery systems. Their RTES based data center cooling system reduced annual electricity consumption by 78% and avoided up to 1488 metric tons of CO_2_‐equivalent emissions annually (Oh et al. 2025). The system also achieved reductions in peak electricity consumption, demonstrating the suitability of ATES‐based solutions for data center cooling under U.S. climatic and subsurface conditions (Oh et al. 2025). The U.S. Geological Survey (USGS), in collaboration with the U.S. Department of Energy (DOE), conducted a national pre‐assessment of a RTES across eight metropolitan areas spanning five geologic regions and diverse climatic conditions in the United States, found RTES to be technically viable in all the locations evaluated (Pepin et al. 2021, 2025).

To simply illustrate the potential performance using an ideal case, consider a mid‐sized data center requiring 5 MW of continuous cooling that consumes 43.8 GWh of thermal energy per year (5 MW for 8760 h/year). Conventional air‐cooled chillers operating with a COP of nearly 3 would provide an annual cooling load equaling 14.6 GWh of electricity. With ATES systems, which use groundwater cooling, the COP could be raised significantly, reaching greater than 10 (Barrios Rivero 2015; Leindals et al. 2024). This efficiency increase would lead to an overall reduction in electricity demand for cooling to as low as 4.4 GWh/year, a nearly 70% reduction in electricity usage compared with the conventional systems. Although this simple calculation with perfect conditions might not be achievable with current practice and technologies, even taking only half of such efficiency (35%) would demonstrate the significant potential of ATES. When ATES is applied in direct cooling operation, system performance can be further enhanced, as cooling is delivered without the use of heat pumps (Jackson et al. 2024). This leads to higher system COP and additional reductions in electricity consumption. When scaled to hundreds of data centers across the United States, the energy efficiency gains would equal hundreds of megawatt‐hours leading to the reduction of tens of thousands of metric tons of greenhouse gas emissions annually (Ramos‐Escudero and Bloemendal 2022).

## Challenges to ATES Adoption for Data Center Cooling in the United States

Despite demonstrated technical feasibility, economic and institutional barriers continue to limit widespread ATES deployment in the United States. European projects have achieved competitive levelized costs of heat in the range of approximately USD 2–9 per MWh_th_ (megawatt‐hour of delivered thermal energy), with wells and subsurface infrastructure accounting for up to 50% of total capital expenditures, and seasonal efficiencies exceeding 60% when hybridized with solar thermal systems (Ghodeswar 2025). In contrast, U.S. projects face higher drilling costs, fragmented regulatory frameworks, and limited financial incentives (Ghodeswar 2025). Nevertheless, the United States possesses extensive favorable geological resources and an existing well infrastructure (e.g., abandoned oil and gas wells) that could support large‐scale ATES deployment if supported by appropriate policy frameworks and financial mechanisms (Ghodeswar 2025).

Technical challenges associated with ATES implementation include limited availability of detailed geological data, subsurface characterization uncertainties, groundwater chemistry issues such as mineral scaling, fouling, and corrosion, risks of aquifer cross‐contamination, thermal imbalance between stored heat and cold, and construction and maintenance complexities (Duijff et al. 2023; García‐Gil et al. 2016; Hähnlein et al. 2013; Liu et al. 2025). In ATES systems involving heat storage, commonly reported challenges include well clogging and clay swelling (Molz et al. 1981, 1983a, 1983b; Holm et al. 1987; Fleuchaus et al. 2018).

Evidence from existing studies suggests that ATES deployment for data center application is effective when integrated within broader energy systems, such as DH networks or hybrid cooling configurations. Accordingly, in the U.S. context, dedicated studies are needed to evaluate thermal management strategies designed around existing and future data center infrastructure to assess whether ATES can meet growing cooling demands. Aquifer characteristics vary considerably across the United States, but systematic comparative assessments across different climatic and hydrogeological settings remain limited. Furthermore, lessons from established European deployments may provide valuable guidance for U.S. applications. For example, aquifers with characteristics similar to the productive sedimentary formations in the Netherlands are found in Coastal Lowlands along the Gulf and Atlantic coasts (USGS 2022). These similarities offer opportunities for knowledge transfer. Also, the spatial alignment between data center development and favorable subsurface conditions is an important planning consideration. Identifying locations where data center cooling demand coincides with suitable aquifer properties, along with opportunities for effective heat recovery, will be essential for the successful deployment of ATES for data centers in the United States.

Beyond technical and regulatory requirements, the deployment of ATES systems is also constrained by market‐related barriers. These include limited awareness and practical experience among mechanical and energy engineers, heating and cooling system installers, and data center designers and owners regarding ATES integration, system operation, and long‐term performance. In addition, investment decisions for building heating and cooling infrastructure tend to favor conventional technologies with well‐established capital and operational cost structures. As a result, emerging geothermal solutions such as ATES are often overlooked in early project planning, despite their potential energy efficiency and operational benefits.

## Conclusion

ATES systems stand out as a feasible and highly scalable heating and cooling technology that is uniquely suited to the evolving cooling needs of data centers. Such systems offer a distinct opportunity to substantially reduce the electricity consumption of data centers and the emission of greenhouse gases while enhancing the operational performance of data centers. ATES is not only a technical upgrade but also a strategic opportunity for the data center market in the United States to lead in energy conservation innovations that provide a level of security in response to perturbations affecting the energy grid. With the demonstrated success of ATES systems in Europe, the case for widespread adoption in the United States is both compelling and timely.

This article highlights that ATES is a mature underground energy storage solution with decades of operational experience, well‐established design principles, and a growing body of performance data. Evidence from European deployments and U.S. demonstration projects indicates that, under suitable hydrogeological and regulatory conditions, ATES can deliver substantial reductions in cooling‐related electricity demand, mitigate peak power loads, enable waste heat recycling, reduce CO_2_ emissions, and significantly reduce water consumption compared to conventional data center cooling technologies.

The integration of ATES systems into data centers can significantly elevate the functional value of groundwater, transforming it from a passive resource into an active component of energy management. This paradigm shift warrants a redefinition of ‘groundwater resources’ to encompass their role in renewable thermal energy storage and thermal regulation, particularly in data center operations and other energy‐intensive AI development.

## Data Availability

Data sharing does not apply to this article as no datasets were generated or analyzed during the current study.
